# Assessing the agreement of biomarker data in the presence of left-censoring

**DOI:** 10.1186/1471-2369-15-144

**Published:** 2014-09-03

**Authors:** Uthumporn Domthong, Chirag R Parikh, Paul L Kimmel, Vernon M Chinchilli

**Affiliations:** 1 Department of Public Health Sciences, Penn State College of Medicine, Hershey, PA, USA; 2 Section of Nephrology, Department of Medicine, Yale University School of Medicine, New Haven, CT, USA; 3 Program of Applied Translational Research, Yale University School of Medicine, New Haven, CT, USA; 4 Division of Kidney Urologic and Hematologic Diseases, NIDDK NIH, Bethesda, MD, USA

## Abstract

**Background:**

In many clinical biomarker studies, Lin’s concordance correlation coefficient (CCC) is commonly used to assess the level of agreement of a biomarker measured under two different conditions. However, measurement of a specific biomarker typically cannot provide accurate numerical values below the lower limit of detection (LLD) of the assay, which results in left-censored data. Most researchers discard the data below the LLD or apply simple data imputation methods in the presence of left-censored data, such as replacing values below the LLD with a fixed number less than or equal to the LLD. This is not statistically optimal, because it often leads to biased estimates and overestimates the precision.

**Methods:**

We describe a simple method using a bivariate normal distribution in this situation and apply SAS statistical software to arrive at the maximum likelihood (ML) estimate of the parameters and construct the estimate of the CCC. We conduct a computer simulation study to investigate the statistical properties of the ML method versus the data deletion and simple data imputation method. We also contrast the methods with real data using two urine biomarkers, Interleukin 18 and Cystatin C.

**Results:**

The computer simulation studies confirm that the ML procedure is superior to the data deletion and simple data imputation procedures. In all of the simulated scenarios, the ML method yields the smallest relative bias and the highest percentage of the 95% confidence intervals that include the true value of the CCC. In the first simulation scenario (sample size of 100 paired data points, 25% left-censoring for both members of the pair, true CCC of 0.238), the relative bias is −1.43% for the ML method, −40.97% for the data deletion method, and it ranges between −12.94% and −21.72% for the simple data imputation methods. Similarly, when the left-censoring for one of the members of the data pairs increases from 25% to 40%, the relative bias displays the same pattern for all methods.

**Conclusions:**

When estimating the CCC from paired biomarker data in the presence of left-censored values, the ML method works better than data deletion and simple data imputation methods.

## Background

Biomarkers in blood and urine are important indicators for the diagnosis of diseases and risk-stratification. During the development of biomarker assays, several pre-analytic steps require comparison of paired values of biomarkers exposed to separate conditions, such as varying degrees of storage, freeze-thaw cycles, and different antibodies. For these experiments, comparison of paired biomarker values is a critical step to advance the development to the next stage. In practice, assays often have lower limits of detection (LLD) due to the limitation of analytic procedures, thereby making the comparison of paired values challenging. A data point below the detection limit is equivalent to being left censored because the exact value of the data point is unknown – it only is known that it lies below the LLD. Although left-censored data are more informative than missing data, they still lead to challenges in the data analysis.

Simple (ad hoc) approaches to address the left-censored data are to delete the value below the LLD or impute a fixed value such as one-half of the LLD or the LLD itself. However, these approaches yield biased estimates of the parameters of interest and they underestimate the variability in the data set because the same value is imputed repeatedly [1-3]. Urine biomarkers are very prone to this problem as the concentration of the biomarkers is greatly influenced by urine volume. In diluted urine, biomarker values may be below the LLD. Also, biomarkers whose concentrations are below ng levels are prone to this problem of having values below LLD. For example, IL-18 is measured in pg/vol in urine, and thus usually has higher proportions of values below the LLD compared to other biomarkers. Many researchers have stressed the importance of data that are below the LLD [1-4].

From the statistical point of view, we can expect the ad hoc methods (data deletion or simple imputation) to estimate the data differently and in a biased manner from the ML approach. An ideal approach to handle the left-censored data is to invoke the ML method because it accounts for the distribution of data in the detectable range and extrapolates into the region below the LLD.

The aim of this study is to show that when faced with left-censored data, the ML approach based on a bivariate normal (or lognormal) assumption for estimating the CCC between two assays is a more appropriate approach to use in practice than the ad hoc approaches that involve data deletion or simple data imputation.

## Results

### Simulation

In Tables 1, 2, 3, 4, 5 and 6, we report the results of a simulation study to assess the means and the standard deviations for estimating the CCC based on the ML approach and compare them to the four different methods that are described in the methods section, which frequently are applied in clinical research. In addition to means and standard deviations, we also report the relative bias, the mean of the standard error, and the percentage of 95% confidence intervals (CI) that include the true value of CCC for the 1,000 simulated data sets.

**Table 1:** Simulation results based on 1000 data sets with sample size of 100 -- Per cent censoring (25%, 25%)

**Per cent censoring**	**True *ρ***	**True *ρ***_***c***_	**Method**	**Mean **${\hat{\rho }}_{c}$	**Relative bias (%)**	**Empirical SD**	**Mean SE**	**The percentage of 95% confidence intervals that include true value of CCC**
(25%, 25%)	0.25	0.238	1. Delete the pair	0.1405	−40.97	0.1119	0.1087	85.2
			2. Replace by LOD	0.2072	−12.94	0.1214	0.0856	91.0
			3. Replace by 0.5 × *LOD*	0.1863	−21.72	0.1630	0.0848	88.2
			4. Replace by *c* × *LOD*	0.1951	−18.03	0.1240	0.0851	89.0
			**5. ML**	**0.2346**	**−1.43**	**0.0944**	**0.0939**	**94.3**
	0.50	0.476	1. Delete the pair	0.2966	−37.69	0.1197	0.0999	61.5
			2. Replace by LOD	0.4169	−12.42	0.1569	0.0735	86.4
			3. Replace by 0.5 × *LOD*	0.3664	−23.03	0.3516	0.0742	78.0
			4. Replace by *c* × *LOD*	0.3821	−19.73	0.1541	0.0744	78.3
			**5. ML**	**0.4701**	**−1.24**	**0.0807**	**0.0780**	**93.6**
	0.75	0.714	1. Delete the pair	0.5307	−25.67	0.0886	0.0762	38.6
			2. Replace by LOD	0.6582	−7.82	0.0996	0.0497	85.1
			3. Replace by 0.5 × *LOD*	0.6140	−14.01	0.1482	0.0519	71.5
			4. Replace by *c* × *LOD*	0.6140	−14.01	0.1662	0.0521	72.1
			**5. ML**	**0.7077**	**−0.88**	**0.0525**	**0.0503**	**94.8**

*Abbreviations: LLD* Lower Limit of Detection, *ML* Maximum Likelihood, C = Random number from uniform distribution (0, 1), *CI* confidence interval, Relative bias = $\frac{\left(\mathrm{Mean}\;{\hat{\rho }}_{c}-\mathrm{True}\;\rho_{c}\right)}{\mathrm{True}\;\rho_{c}}\times 100\%$, CCC^5^= $\rho_{c}=\frac{2\mathrm{COV}\left(X,Y\right)}{\mathrm{VAR}\left(X\right)+\mathrm{VAR}\left(Y\right)+{\left(E\left(X\right)-E\left(Y\right)\right)}^{2}}$.

**Table 2:** Simulation results based on 1000 data sets with sample size of 100 -- Per cent censoring (40%, 25%)

**Per cent censoring**	**True *ρ***	**True *ρ***_***c***_	**Method**	**Mean **${\hat{\rho }}_{c}$	**Relative bias (%)**	**Empirical SD**	**Mean SE**	**The percentage of 95% confidence intervals that include true value of CCC**
(40%, 25%)	0.25	0.238	1. Delete the pair	0.1240	−47.90	0.1279	0.1230	84.9
			2. Replace by LOD	0.1929	−18.95	0.1319	0.0843	86.8
			3. Replace by 0.5 × *LOD*	0.1856	−22.02	0.1092	0.0845	85.9
			4. Replace by *c* × *LOD*	0.1850	−22.27	0.1180	0.0844	85.7
			**5. ML**	**0.2340**	**−1.68**	**0.0984**	**0.0971**	**93.9**
	0.50	0.476	1. Delete the pair	0.2754	−42.14	0.1190	0.1108	60.2
			2. Replace by LOD	0.3901	−18.05	0.1219	0.0727	80.1
			3. Replace by 0.5 × *LOD*	0.3661	−23.09	0.1488	0.0736	73.7
			4. Replace by *c* × *LOD*	0.3664	−23.03	0.1603	0.0735	74.0
			**5. ML**	**0.4692**	**−1.43**	**0.0847**	**0.0811**	**93.8**
	0.75	0.714	1. Delete the pair	0.4966	−30.45	0.0978	0.0851	30.7
			2. Replace by LOD	0.6268	−12.21	0.1054	0.0500	68.7
			3. Replace by 0.5 × *LOD*	0.6055	−15.20	0.1389	0.0512	64.7
			4. Replace by *c* × *LOD*	0.6083	−14.80	0.1306	0.0513	65.0
			**5. ML**	**0.7066**	**−1.04**	**0.0554**	**0.0528**	**94.8**

*Abbreviations: LLD* Lower Limit of Detection, *ML* Maximum Likelihood, C = Random number from uniform distribution (0, 1), *CI*, confidence interval, Relative bias = $\frac{\left(\mathrm{Mean}\;{\hat{\rho }}_{c}-\mathrm{True}\;\rho_{c}\right)}{\mathrm{True}\;\rho_{c}}\times 100\%$, CCC^5^= $\rho_{c}=\frac{2\mathrm{COV}\left(X,Y\right)}{\mathrm{VAR}\left(X\right)+\mathrm{VAR}\left(Y\right)+{\left(E\left(X\right)-E\left(Y\right)\right)}^{2}}$.

**Table 3:** Simulation results based on 1000 data sets with sample size of 50 -- Per cent censoring (25%, 25%)

**Per cent censoring**	**True *ρ***	**True *ρ***_***c***_	**Method**	**Mean **${\hat{\rho }}_{c}$	**Relative bias (%)**	**Empirical SD**	**Mean SE**	**The percentage of 95% confidence intervals that include true value of CCC**
(25%, 25%)	0.25	0.238	1. Delete the pair	0.1402	−41.09	0.1693	0.1486	87.1
			2. Replace by LOD	0.2079	−12.65	0.1509	0.1172	88.2
			3. Replace by 0.5 × *LOD*	0.1811	−23.91	0.2088	0.1163	83.8
			4. Replace by *c* × *LOD*	0.1949	−18.09	0.1813	0.1162	83.9
			**5. ML**	**0.2310**	**−2.94**	**0.1351**	**0.1304**	**92.7**
	0.50	0.476	1. Delete the pair	0.2936	−38.32	0.1481	0.1386	79.4
			2. Replace by LOD	0.4144	−12.94	0.4115	0.1016	87.3
			3. Replace by 0.5 × *LOD*	0.3661	−23.09	0.1854	0.1024	79.4
			4. Replace by *c* × *LOD*	0.3722	−21.81	0.1770	0.1026	81.2
			**5. ML**	**0.4636**	**−2.61**	**0.1151**	**0.1095**	**93.4**
	0.75	0.714	1. Delete the pair	0.5169	−27.61	0.1341	0.1071	66.7
			2. Replace by LOD	0.6429	−9.96	0.1438	0.0694	88.3
			3. Replace by 0.5 × *LOD*	0.5812	−18.60	0.2127	0.0720	78.5
			4. Replace by *c* × *LOD*	0.5909	−17.24	0.1917	0.0727	79.6
			**5. ML**	**0.7025**	**−1.61**	**0.0759**	**0.0716**	**93.6**

*Abbreviations: LLD* Lower Limit of Detection; *ML* Maximum Likelihood, C = Random number from uniform distribution (0, 1), *CI* confidence interval, Relative bias = $\frac{\left(\mathrm{Mean}\;{\hat{\rho }}_{c}-\mathrm{True}\;\rho_{c}\right)}{\mathrm{True}\;\rho_{c}}\times 100\%$ CCC^5^= $\rho_{c}=\frac{2\mathrm{COV}\left(X,Y\right)}{\mathrm{VAR}\left(X\right)+\mathrm{VAR}\left(Y\right)+{\left(E\left(X\right)-E\left(Y\right)\right)}^{2}}$.

**Table 4:** Simulation results based on 1000 data sets with sample size of 50 -- Per cent censoring (40%, 25%)

**Per cent censoring**	**True *ρ***	**True *ρ***_***c***_	**Method**	**Mean **${\hat{\rho }}_{c}$	**Relative bias (%)**	**Empirical SD**	**Mean SE**	**The percentage of 95% confidence intervals that include true value of CCC**
(40%, 25%)	0.25	0.238	1. Delete the pair	0.1334	−43.95	0.1858	0.1652	88.1
			2. Replace by LOD	0.1908	−19.83	0.1525	0.1150	84.3
			3. Replace by 0.5 × *LOD*	0.1796	−24.54	0.1467	0.1155	80.2
			4. Replace by *c* × *LOD*	0.1780	−25.22	0.1528	0.1152	81.8
			**5. ML**	**0.2296**	**−3.53**	**0.1395**	**0.1345**	**92.4**
	0.50	0.476	1. Delete the pair	0.2735	−42.54	0.1693	0.1516	76.9
			2. Replace by LOD	0.3673	−22.84	0.2110	0.1004	80.2
			3. Replace by 0.5 × *LOD*	0.3366	−29.29	0.2771	0.1011	74.3
			4. Replace by *c* × *LOD*	0.3537	−25.70	0.1788	0.1011	75.9
			**5. ML**	**0.4617**	**−3.00**	**0.1206**	**0.1135**	**93.2**
	0.75	0.714	1. Delete the pair	0.4913	−31.19	0.1373	0.1184	62.3
			2. Replace by LOD	0.6022	−15.66	0.1705	0.0696	78.7
			3. Replace by 0.5 × *LOD*	0.5706	−20.08	0.2039	0.0710	74.7
			4. Replace by *c* × *LOD*	0.5689	−20.32	0.2151	0.0713	74.1
			**5. ML**	**0.7003**	**−1.92**	**0.0805**	**0.0750**	**93.2**

*Abbreviations: LLD* Lower Limit of Detection, *ML* Maximum Likelihood, C = Random number from uniform distribution (0, 1), *CI* confidence interval, Relative bias = $\frac{\left(\mathrm{Mean}\;{\hat{\rho }}_{c}-\mathrm{True}\;\rho_{c}\right)}{\mathrm{True}\;\rho_{c}}\times 100\%$, CCC^5^= $\rho_{c}=\frac{2\mathrm{COV}\left(X,Y\right)}{\mathrm{VAR}\left(X\right)+\mathrm{VAR}\left(Y\right)+{\left(E\left(X\right)-E\left(Y\right)\right)}^{2}}$.

**Table 5:** Simulation results based on 1000 data sets with sample size of 25 -- Per cent censoring (25%, 25%)

**Per cent censoring**	**True *ρ***	**True *ρ***_***c***_	**Method**	**Mean **${\hat{\rho }}_{c}$	**Relative bias (%)**	**Empirical SD**	**Mean SE**	**The percentage of 95% confidence intervals that include true value of CCC**
(25%, 25%)	0.25	0.238	1. Delete the pair	0.1300	−45.38	0.2287	0.2002	85.7
			2. Replace by LOD	0.2159	−9.29	0.2176	0.1563	84.1
			3. Replace by 0.5 × *LOD*	0.1821	−23.49	0.2280	0.1550	78.5
			4. Replace by *c* × *LOD*	0.1897	−20.29	0.2215	0.1552	78.4
			**5. ML**	**0.2225**	**−6.51**	**0.1905**	**0.1783**	**93.1**
	0.50	0.476	1. Delete the pair	0.2862	−39.87	0.2088	0.1853	83.4
			2. Replace by LOD	0.3963	−16.74	0.2030	0.1356	83.1
			3. Replace by 0.5 × *LOD*	0.3538	−25.67	0.2277	0.1372	76.3
			4. Replace by *c* × *LOD*	0.3642	−23.49	0.2105	0.1372	78.7
			**5. ML**	**0.4496**	**−5.55**	**0.1650**	**0.1526**	**93.3**
	0.75	0.714	1. Delete the pair	0.4850	−32.07	0.1951	0.1489	77.3
			2. Replace by LOD	0.6031	−15.53	0.2122	0.0960	83.5
			3. Replace by 0.5 × *LOD*	0.5467	−23.43	0.2545	0.0984	77.0
			4. Replace by *c* × *LOD*	0.5436	−23.87	0.2700	0.0991	77.6
			**5. ML**	**0.6897**	**−3.40**	**0.1136**	**0.1027**	**92.1**

*Abbreviations: LLD* Lower Limit of Detection, *ML* Maximum Likelihood, C = Random number from uniform distribution (0, 1), *CI* confidence interval, Relative bias = $\frac{\left(\mathrm{Mean}\;{\hat{\rho }}_{c}-\mathrm{True}\;\rho_{c}\right)}{\mathrm{True}\;\rho_{c}}\times 100\%$, CCC^5^= $\rho_{c}=\frac{2\mathrm{COV}\left(X,Y\right)}{\mathrm{VAR}\left(X\right)+\mathrm{VAR}\left(Y\right)+{\left(E\left(X\right)-E\left(Y\right)\right)}^{2}}$.

**Table 6:** Simulation results based on 1000 data sets with sample size of 25 -- Per cent censoring (40%, 25%)

**Per cent censoring**	**True *ρ***	**True *ρ***_***c***_	**Method**	**Mean **${\hat{\rho }}_{c}$	**Relative bias (%)**	**Empirical SD**	**Mean SE**	**The percentage of 95% confidence intervals that include true value of CCC**
(40%, 25%)	0.25	0.238	1. Delete the pair	0.1357	−42.98	0.2485	0.2130	85.5
			2. Replace by LOD	0.1703	−28.45	0.4223	0.1530	80.1
			3. Replace by 0.5 × *LOD*	0.1822	−23.45	0.2936	0.1535	74.3
			4. Replace by *c* × *LOD*	0.1751	−26.45	0.2033	0.1533	75.4
			**5. ML**	**0.2193**	**−7.86**	**0.1948**	**0.1824**	**93.0**
	0.50	0.476	1. Delete the pair	0.2667	−43.97	0.2296	0.1982	82.3
			2. Replace by LOD	0.3628	−23.78	0.2088	0.1334	78.6
			3. Replace by 0.5 × *LOD*	0.3307	−30.53	0.2283	0.1350	71.7
			4. Replace by *c* × *LOD*	0.3304	−30.59	0.2416	0.1352	72.8
			**5. ML**	**0.4449**	**−6.53**	**0.1693**	**0.1570**	**93.3**
	0.75	0.714	1. Delete the pair	0.4600	−35.57	0.2050	0.1608	75.9
			2. Replace by LOD	0.5599	−21.58	0.2482	0.0947	78.4
			3. Replace by 0.5 × *LOD*	0.5246	−26.53	0.2619	0.0961	72.8
			4. Replace by *c* × *LOD*	0.5266	−26.25	0.2678	0.0964	74.0
			**5. ML**	**0.6847**	**−4.10**	**0.1180**	**0.1074**	**92.2**

*Abbreviations: LLD* Lower Limit of Detection, *ML* Maximum Likelihood, C = Random number from uniform distribution (0, 1), *CI* confidence interval, Relative bias = $\frac{\left(\mathrm{Mean}\;{\hat{\rho }}_{c}-\mathrm{True}\;\rho_{c}\right)}{\mathrm{True}\;\rho_{c}}\times 100\%$, CCC^5^= $$\rho_{c}=\frac{2\mathrm{COV}\left(X,Y\right)}{\mathrm{VAR}\left(X\right)+\mathrm{VAR}\left(Y\right)+{\left(E\left(X\right)-E\left(Y\right)\right)}^{2}}$$.

As demonstrated in Tables 1, and 2, the estimates from the four simple approaches are obviously biased, although the replacement of non-detectable data by a fraction of the detection limit or the detection limit itself is clearly preferable to discarding the pair method for all range of sample sizes. From Table 1 (sample size of 100 paired data points, 25% left-censoring for X, 25% left-censoring for Y, and a true CCC of 0.238), the relative bias is −1.43% for the ML method, −40.97% for the data deletion method, and it ranges between −12.94% and −21.72% for the simple data imputation methods. These four *ad hoc* methods also overstate the precision by underestimating the standard error set because the same value is imputed repeatedly. As expected, the ML method provides an excellent estimate of the true value of the CCC even when the censoring percentages increased, but it tends to slightly underestimate the true value. Moreover, the ML approach yields the smallest relative bias and the highest percentage of the 95% CI that include the true value of CCC among five methods. To see the impact of the percent of censoring, in Table 2, we increase the censoring rate to 40%. The relative biases are increased in all approaches. However, the ML approach still yields the smallest relative bias. From Table 2 (sample size of 100 paired data points, 40% left-censoring for X, 25% left-censoring for Y, and a true CCC of 0.238), the relative bias is −1.68% for the ML method, −47.90% for the data deletion method, and it ranges between −18.95% and −22.27% for the simple data imputation methods. The ML method also has the highest percentage of confidence intervals that include the true value of the CCC.

Due to the large sample size of both assays (sample size =100) in Tables 1 and 2, the ML method displays an excellent result for estimating the CCC with respect to the relative bias and the percentage of confidence intervals that include the true value of the CCC. However, if the sample size were smaller, then the ML method might produce less convincing results. To illustrate this point, we re-conduct the simulation studies with sample sizes = 50 (Tables 3 and 4) and sample sizes = 25 (Tables 5 and 6). In both cases, the ML method still performs best among the five approaches according to the means and the standard deviations for estimating the CCC, the relative bias, mean of the standard error, and the percentage of 95% CI that include the true value of CCC.

### Example

We illustrate these issues further via a urine stability study to assess agreement for two assays with lower limits of detection. The data set came from the multi-center ASSESS AKI Study (the Assessment, Serial Evaluation, and Subsequent Sequelae of Acute Kidney Injury). The data set was originally analyzed by Parikh *et al.*[4]. The purpose of the ASSESS AKI sub-study was to determine the agreement between the measurements of the urinary biomarkers collected under a standard condition and under different experimental conditions, denoted as Process A, Process B, and Process C. Each experimental situation consisted of 50 paired samples (a selected process versus the standard). There are two biomarkers that we consider here: urine Interleukin 18 (IL-18; LLD = 12.5 pg/ ml), and urine Cystatin C (LLD = 0.005 mg/ml). The IL-18 contained 99 undetectable readings (out of a total of 300), yielding a 33% left-censoring rate. The Cystatin C contained 80 undetectable readings, for a 26.7% left-censoring rate. A natural logarithm transformation was applied to both the IL-18 and Cystatin C readings. We treat the natural logarithm of Process A, Process B, and Process C as the X variable and the natural logarithm of the reference standard as the Y variable.

Tables 7 and 8 summarize the results based on the four *ad hoc* approaches and the ML method, for estimating the CCC when comparing the reference standard process to Process A, Process B, and Process C for IL-18 and Cystatin C, respectively. As this example suggests, the four simple approaches can lead to CCC estimates that are different than the CCC estimated from the ML method. For example, from comparing Process B to the standard for urine IL-18 in Table 7, the CCC estimate is 0.73 from the data deletion method, 0.61 from each of the simple data imputation methods, and 0.68 from the ML method.

**Table 7:** Concordance correlation coefficients (and 95% confidence intervals) for 3 processes using 5 methods based on IL-18* assay

**Processes**	**Method 1**	**Method 2**	**Method 3**	**Method 4**	**Method 5**
	**Delete the pair**	**Replace by LOD**	**Replace by 0.5 × *LOD***	**Replace by *c *** **× *LOD***	**ML**
**A (Initial 48 hours: 4°C vs −80°C)**	0.8801 (0.81, 0.95)	0.8228 (0.73, 0.91)	0.8228 (0.73, 0.91)	0.8228 (0.73, 0.91)	0.8314 (0.74, 0.92)
**B (Initial 48 hours: 25°C vs −80°C)**	0.7344 (0.56, 0.91)	0.6081 (0.43, 0.77)	0.6081 (0.43, 0.77)	0.6081 (0.43, 0.77)	0.6819 (0.51, 0.85)
**C (Centrifuge vs No Centrifuge)**	0.9886 (0.98, 1.00)	0.9896 (0.98, 1.00)	0.9896 (0.98, 1.00)	0.9896 (0.98, 1.00)	0.9876 (0.99, 1.00)

*IL-18 (Interleukin 18).

**Table 8:** Concordance correlation coefficients (and 95% confidence intervals) for 3 processes using 5 methods based on Cystatin C assay

**Processes**	**Method 1**	**Method 2**	**Method 3**	**Method 4**	**Method 5**
	**Delete the pair**	**Replace by LOD**	**Replace by 0.5 × *LOD***	**Replace by *c *** **× *LOD***	**ML**
**A (Initial 48 hours: 4°C vs- 80°C)**	0.9348 (0.89, 0.98)	0.9641 (0.94, 0.98)	0.9641 (0.94, 0.98)	0.9641 (0.94, 0.98)	0.9735 (0.95, 0.99)
**B (Initial 48 hours: 25°C vs −80°C)**	0.9320 (0.89, 0.98)	0.9514 (0.92, 0.98)	0.9514 (0.92, 0.98)	0.9514 (0.92, 0.98)	0.9471 (0.91, 0.98)
**C (Centrifuge vs No Centrifuge)**	0.9985 (0.99, 1.00)	0.9982 (0.99, 1.00)	0.9758 (0.96, 0.99)	0.9653 (0.95, 0.98)	0.9999 (0.99, 1.00)

## Discussion

Biomarkers are being discovered at an accelerated rate due to availability of genomic and proteomic technologies [5]. Several of these candidate biomarkers are undergoing validation to diagnose diseases and serve as indices for predicting health outcomes. The main purpose of our study was to assist the biomarker development program by confirming that the simple data imputation approaches and the deletion of data are not optimal techniques for arriving at accurate (unbiased) results with the appropriate level of precision in the presence of left-censored data.

Many researchers have stressed the importance of data that are below the LLD [1-4]. Hornung and Reed [1] proposed three methods of estimation with a left-censored lognormal distribution: a maximum likelihood (ML) method and two methods involving the limit of detection. However, they conclude that the ML method is complex to calculate, so they recommend using the one-half of the LLD. Lyles *et al.*[2] evaluated the Pearson’s correlation coefficient when a subset of data points was below the LLD by using the ML approach under the assumption of bivariate normality. They showed that the ML method was the most accurate among the proposed methods. Barnhart *et al*. [3] presented a generalized estimating equations (GEE) approach for estimating parameters to calculate the concordance correlation coefficient (CCC) [6], which is a measure of agreement ranging between −1 and +1 for paired data. The GEE approach works well and does not require the bivariate normality assumption if the sample size is large enough, and it is comparable to the ML approach when the bivariate normality assumption is appropriate. Parikh *et al*. [4] performed a prospective study on hospitalized patients with almost 60% of patients having acute kidney injury (AKI). Five urine biomarkers were used to compare the stability of short-term storage and processing by using the CCC as a measure of agreement. To estimate the CCC, the authors applied the ML method using log-transformed data and accounting for values below the LLD.

We have illustrated with our computer simulation study that the estimation of the CCC from the imputation methods or data deletion lead to biased estimates compared to the ML approach. We also have shown via the computer simulation study that the proportion of left-censored data significantly impacts the degree of bias in estimating the CCC. Our simulation study shows that the ML approach based on the bivariate normality assumption works best among all of the studied approaches. The advantages of the ML approach are that it is accurate (small relative bias) and accounts for the variability in the data set appropriately. Additionally, it uses all the available data for the statistical analysis, in contrast to the data deletion approach that only uses sample pairs with both values above the LLD in the analysis. The estimates from the data deletion approach are obviously biased and result in a (1) large relative bias and (2) a high value of the standard error due to a small sample size from deleting paired data points. Although assigning a fixed value such as the LLD (or one-half of the LLD or the multiplication of the LLD by a random number from the uniform(0,1) distribution), yields smaller relative biases compared to the data deletion approach, the precision from these methods is overestimated due to the assignment of the same value to data below the LLD.

Although we did not investigate the performance of the ML method for censoring above 40%, we expect that the ML method still will perform well when censoring exceeds 50%. Lyles *et al.*[2] investigated 60% censoring for their situation and the ML method still maintained a high level of accuracy.

## Conclusions

The ML approach is very accurate in that it yields small relative biases if the assumption of bivariate normality is appropriate, and it can be readily implemented using SAS PROC NLMIXED [see Additional file 1 for a sample program]. Thus, our simulation study suggests that the ML approach is best for biomarker assay development where paired results need to be compared.

## Methods

To find the optimal method to deal with left-censored data, we investigate how data deletion and simple data imputation methods compare to the ML approach in a computer simulation study. We adapt the framework from Barnhart *et al.*[3] for our computer simulation studies. In all simulations described in the results, we generate bivariate normal data for paired data represented by the variables *X* and *Y* with a sample size of 100, 50, 25 for each of 1000 data sets using one of the following six combinations of parameter settings for the means, standard deviations, and correlation coefficient: *μ*_*x*_ = 0, *μ*_*y*_ = 0.2, *σ*_*x*_ = 0.8, *σ*_*y*_ = 1, *ρ* = 0.25, 0.50, 0.75, and left-censoring rates of (25% for X, 25% for Y) or (40% for X, 25% for Y). The selected values of the LLDs in the simulation study are determined by the censoring rates. All calculations are performed using SAS 9.3 statistical software. All estimated CCCs (${\hat{\rho }}_{c}$) were obtained by maximizing the likelihood function with respect to each of the following five scenarios.

1. Deleting the pair method means that pairs with X, Y, or both X and Y below the detection limit are discarded before calculation of the CCC. The 95% confidence interval (CI) of this method is calculated by using ${\hat{\rho }}_{c}\pm Z_{0.025}\mathrm{SE}\left({\hat{\rho }}_{c}\right)$ where ${\hat{\rho }}_{c}$ is the estimated CCC, *Z*_0.025_ is the critical value of the standard normal distribution, and $\mathrm{SE}\left({\hat{\rho }}_{c}\right)$ is the standard error of the estimated CCC.

2. Replacing the left-censored data by the LLD method refers to the use of the CCC after replacing all non-detectable data by the applicable detection limit. The 95% confidence interval (CI) of this method is calculated by using ${\hat{\rho }}_{c}\pm Z_{0.025}\mathrm{SE}\left({\hat{\rho }}_{c}\right)$ where ${\hat{\rho }}_{c}$ is the estimated CCC, *Z*_0.025_ is the critical value of the standard normal distribution, and $\mathrm{SE}\left({\hat{\rho }}_{c}\right)$ is the standard error of the estimated CCC.

3. Replacing the left-censored data by one-half of the LLD method refers to the calculation of the CCC using all pairs after replacing the non-detectable data with 0.5 times the detection limit. The 95% confidence interval (CI) of this method is calculated by using ${\hat{\rho }}_{c}\pm Z_{0.025}\mathrm{SE}\left({\hat{\rho }}_{c}\right)$ where ${\hat{\rho }}_{c}$ is the estimated CCC, *Z*_0.025_ is the critical value of the standard normal distribution, and $\mathrm{SE}\left({\hat{\rho }}_{c}\right)$ is the standard error of the estimated CCC.

4. Replacing the left-censored data by c × LLD method refers to the situation in which we first generate a random number from the uniform (0, 1) distribution, say c. Then, we replace each non-detectable data point with c times the detection limit. A new value of c is determined for each non-detectable data point. The 95% confidence interval (CI) of this method is calculated by using ${\hat{\rho }}_{c}\pm Z_{0.025}\mathrm{SE}\left({\hat{\rho }}_{c}\right)$ where ${\hat{\rho }}_{c}$ is the estimated CCC, *Z*_0.025_ is the critical value of the standard normal distribution, and $\mathrm{SE}\left({\hat{\rho }}_{c}\right)$ is the standard error of the estimated CCC.

5. The ML approach is performed by constructing a likelihood function based on the bivariate normal distribution of the data in the detectable range, and then extrapolating into the region below the LLD. The 95% confidence interval (CI) of this method is calculated by using ${\hat{\rho }}_{c}\pm Z_{0.025}\mathrm{SE}\left({\hat{\rho }}_{c}\right)$ where ${\hat{\rho }}_{c}$ is the estimated CCC, *Z*_0.025_ is the critical value of the standard normal distribution, and $\mathrm{SE}\left({\hat{\rho }}_{c}\right)$ is the standard error of the estimated CCC. An additional file displays a sample SAS program for the calculations [see Additional file 1] and another additional file explains this ML approach in more detail [see Additional file 2].

## Competing interests

The authors declare that they have no competing interests.

## Authors’ contributions

UD performed the statistical analysis and drafted the manuscript. CRP and PLK provided the expertise on biomarker kidney studies. VMC conceived of the study, and participated in its design and coordination. All authors read and approved the final manuscript.

## Pre-publication history

The pre-publication history for this paper can be accessed here:

http://www.biomedcentral.com/1471-2369/15/144/prepub

## Supplementary Material

Additional file 1 **SAS program with an example.** The following code is written in SAS 9.3 statistical software to demonstrate how to use the maximum likelihood approach. To run this code, copy the SAS code script below to the Editor window in SAS, and submit it to get the results. Click here for file

Additional file 2 Maximum Likelihood Method. We provide the detail on the Maximum likelihood method. Click here for file
