# Supplementary material for: Assessing the agreement of biomarker data in the presence of left-censoring
Source: BMC Nephrol. 2014 Sep 3;15:144. doi: 10.1186/1471-2369-15-144 (PMC4236661; doi:10.1186/1471-2369-15-144)
Supplement: Additional file 1 — SAS program with an example. The following code is written in SAS 9.3 statistical software to demonstrate how to use the maximum likelihood approach. To run this code, copy the SAS code script below to the Editor window in SAS, and submit it to get the results. [file 1471-2369-15-144-S1.docx]

**Additional file 1 - - Example SAS Program**

The following code is written in SAS 9.3 statistical software to demonstrate how to use the maximum likelihood approach. To run this code, copy the SAS code script below to the Editor window in SAS, and submit it to get the results.

| *************************************************************************************  * This SAS program demonstrates how to apply proc nlmixed to estimate the *  * concordance correlation coefficient under the assumption of bivariate normality *  * for the variables X and Y, where X and Y can be left-censored. *  *************************************************************************************;  *************************************************************************************  * Construct the SAS dataset and assume that the lower limit of detection (LLD_X) of *  * X = -0.55 and the lower limit of detection (LLD_Y) of Y = -0.22). *  *************************************************************************************;  **data** BVN_ML;  input ID X_variable Y_variable;  LLD_X = -**0.55**;  LLD_Y = -**0.22**;  cards;  1 0.99254 -0.00816  2 -0.81115 0.61426  3 -0.25967 -1.54448  4 -0.54144 -2.08703  5 0.31190 0.00612  6 0.60156 -0.41427  7 -0.91292 0.67860  8 0.53728 0.65432  9 0.58839 1.02270  10 0.32547 -1.25067  11 0.38447 0.38117  12 -0.99933 0.11877  13 0.96899 0.25556  14 -0.14445 1.69732  15 0.18829 0.61474  16 1.38879 0.13411  17 0.06209 -0.78281  18 -2.28957 -0.53707  19 0.11634 -0.34868  20 0.25130 0.94796  21 -0.98698 0.25604  22 0.37693 -0.59843  23 -1.48264 0.34309  24 2.47979 1.90679  25 0.01393 -2.20015  26 -0.42399 0.72970  27 1.32877 1.46191  28 0.78614 1.00235  29 -0.25541 -0.68138  30 -0.25670 -0.77916  31 -0.17793 -0.82038  32 -0.87692 0.77897  33 0.90345 1.91300  34 -0.68123 1.13219  35 0.56072 0.99976  36 0.86430 0.27925  37 -0.44175 1.15311  38 0.67694 -0.11090  39 0.91961 1.92702  40 -0.54352 -1.33920  41 -0.67072 -0.78333  42 -0.33217 0.41390  43 -0.86068 1.59898  44 0.14378 1.96710  45 -1.17482 0.54201  46 1.58460 1.03018  47 -0.57426 -1.29707  48 -0.19701 -0.20496  49 0.56686 0.87197  50 0.77084 1.66170  51 0.72289 1.41742  52 -1.26496 0.30734  53 0.17253 0.56950  54 0.20422 -0.00090  55 -1.12339 0.49165  56 -0.18836 -0.05975  57 -0.36461 -0.25222  58 -0.93629 0.41876  59 -0.16318 1.65691  60 -0.56996 -0.05180  61 0.34613 0.48361  62 0.22510 1.87404  63 -0.32070 0.31165  64 0.52935 0.88800  65 0.24909 0.73543  66 0.58197 -0.15928  67 -0.18697 -0.37119  68 -0.23600 -1.66764  69 0.06795 0.46950  70 -0.17071 0.99734  71 -0.68762 0.34101  72 0.08471 -1.57191  73 0.99670 -0.17914  74 -0.29238 0.81303  75 -1.28748 0.34764  76 -0.21653 0.15241  77 0.04435 0.14768  78 -1.15762 -0.05178  79 -1.19074 -0.52243  80 0.40536 1.19296  81 0.69441 0.98494  82 -0.14392 -0.56590  83 1.07026 -0.67580  84 1.10691 0.56373  85 -0.10203 0.07566  86 1.59507 0.42948  87 0.68092 0.90975  88 0.70821 1.30926  89 -0.40952 0.69317  90 -0.47999 -0.05661  91 -0.47487 -0.15166  92 -0.55780 -0.44337  93 -0.67149 0.01235  94 -0.61205 -0.24293  95 -0.61361 1.01447  96 -1.23624 0.27788  97 0.45496 0.46340  98 -0.11075 0.36547  99 0.63039 1.06721  100 1.13935 -0.62838  ;  **run**;  **proc** **nlmixed** data=BVN_ML;  parms mu_X=**0** mu_Y=**0** sigma_XX=**1** sigma_YY=**1** sigma_XY=**0**;  *Case 1: X > LLD_X and Y > LLD_Y;  ll_11_logdet=-**0.5***log(((sigma_XX*sigma_YY)-(sigma_XY*sigma_XY)));  ll_11_quadform=-**0.5***((sigma_YY*(X_variable-mu_X)*(X_variable-mu_X))+(sigma_XX*(Y_variable-mu_Y)*  (Y_variable-mu_Y))-**2***(sigma_XY*(X_variable-mu_X)*(Y_variable-mu_Y)))/  ((sigma_XX*sigma_YY)-(sigma_XY*sigma_XY));  ll_11=ll_11_logdet+ll_11_quadform;  *Case 2: X <= LLD_X and Y > LLD_Y;  ll_01_logX=log(probnorm((LLD_X-mu_X-((Y_variable-mu_Y)*(sigma_XY/sigma_YY)))/  sqrt(((sigma_XX*sigma_YY)-(sigma_XY*sigma_XY))/sigma_YY)));  ll_01_logY=-**0.5***(log(sigma_YY)+((Y_variable-mu_Y)*(Y_variable-mu_Y)/sigma_YY));  ll_01=ll_01_logX+ll_01_logY;  *Case 3: X > LLD_X and Y <= LLD_Y;  ll_10_logY=log(probnorm((LLD_Y-mu_Y-((X_variable-mu_X)*(sigma_XY/sigma_XX)))/  sqrt(((sigma_XX*sigma_YY)-(sigma_XY*sigma_XY))/sigma_XX)));  ll_10_logX=-**0.5***(log(sigma_XX)+((X_variable-mu_X)*(X_variable-mu_X)/sigma_XX));  ll_10=ll_10_logX+ll_10_logY;  *Case 4: X <= LLD_X and Y <= LLD_Y;  ll_00=log(probbnrm((LLD_X-mu_X)/sqrt(sigma_XX),(LLD_Y-mu_Y)/sqrt(sigma_YY),sigma_XY/  sqrt(sigma_XX*sigma_YY)));  *Combine all four cases to get the likelihood function;  ll=((X_variable>LLD_X)*(Y_variable>LLD_Y)*ll_11)+  ((X_variable<=LLD_X)*(Y_variable>LLD_Y)*ll_01)+  ((X_variable>LLD_X)*(Y_variable<=LLD_Y)*ll_10)+  ((X_variable<=LLD_X)*(Y_variable<=LLD_Y)*ll_00);  variable=**0.5***(X_variable+Y_variable);  model variable ~ general(ll);  estimate 'Concordance Correlation' **2***sigma_XY/(sigma_XX+sigma_YY+((mu_x-mu_Y)*(mu_X-mu_Y)));  **run**; |
| --- |
