# Supplementary material for: Assessing the agreement of biomarker data in the presence of left-censoring
Source: BMC Nephrol. 2014 Sep 3;15:144. doi: 10.1186/1471-2369-15-144 (PMC4236661; doi:10.1186/1471-2369-15-144)
Supplement: Additional file 2 — Maximum Likelihood Method. We provide the detail on the Maximum likelihood method. [file 1471-2369-15-144-S2.docx]

**Additional file 2 - - Maximum likelihood Method**

To assess the level of agreement of a biomarker measured under two different conditions, we use Lin’s concordance correlation coefficient (CCC^5^) index, expressed as

$CCC= \rho_{c}=\frac{2COV(X,Y)}{VAR\left( X \right)+VAR\left( Y \right)+\left( E\left( X \right)-E\left( Y \right) \right)^{2}}$ (1)

The maximum likelihood method^3^ (ML method) under a bivariate normality assumption for estimating the CCC in the presence of left-censored data can be obtained by inserting the ML estimates for $COV\left( X,Y \right), VAR\left( X \right),VAR\left( Y \right),E\left( X \right),E\left( Y \right)$ into equation (1). Let ${(x}_{i}, y_{i}), i=1,2,\ldots, N$ denote the random sample from random variables ${(X}_{i}, Y_{i}),$ so that the ML estimates can be obtained by maximizing the observed data likelihood, based on N pairs of ${(X}_{i}, Y_{i})$

$$L= \prod_{i=1}^{N} \left[ BVN(\frac{x_{i}-\mu_{x}}{\sigma_{x}}, \frac{y_{i}-\mu_{y}}{\sigma_{y}}, \sigma_{xy}) \right]^{d_{1i}}\left[ \phi(\frac{x_{i}-\mu_{x}}{\sigma_{x}})\Phi(\frac{\mathrm{LLD}_{y}-\mu_{y|x_{i}}}{\sigma_{y|x_{i}}}) \right]^{d_{2i}}\times\left[ \Phi(\frac{\mathrm{LLD}_{x}-\mu_{x|y_{i}}}{\sigma_{x|y_{i}}})\phi(\frac{y_{i}-\mu_{y}}{\sigma_{y}}) \right]^{d_{3i}}\left[ BVN(\frac{\mathrm{LLD}_{x}-\mu_{x}}{\sigma_{x}}, \frac{\mathrm{LLD}_{y}-\mu_{y}}{\sigma_{y}}, \sigma_{xy}) \right]^{d_{4i}}$$

where BVN, $\phi(.)$ and $\Phi(.)$are the bivariate normal distribution, the standard univariate normal density and cumulative distribution functions, respectively, $\mu_{x},\sigma_{x}$ is the mean of $X$ and the standard deviation of$X$, respectively; $\mu_{y|x_{i}}$ is the conditional mean of $Y$ given$X_{i}$; $\sigma_{y|x_{i}}$ is the conditional standard deviation of $Y$ given$X_{i}$, and $d_{1i}, d_{2i},d_{3i},d_{4i}$are indicator variables for the following four conditions, respectively: (1) $x_{i} \geq\mathrm{LLD}_{x}$ and $y_{i} \geq\mathrm{LLD}_{y}$ (2) $x_{i} \geq\mathrm{LLD}_{x}$ and $y_{i}< \mathrm{LLD}_{x}$ (3) $x_{i}< \mathrm{LLD}_{x}$ and $y_{i}\geq\mathrm{LLD}_{x}$ (4) $x_{i}< \mathrm{LLD}_{x}$ and $y_{i}< \mathrm{LLD}_{x}$.
